# Mechanical Energy Harvesting Performance of Ferroelectric Polymer Nanowires Grown via Template‐Wetting

**DOI:** 10.1002/ente.201700820

**Published:** 2018-02-16

**Authors:** Richard A. Whiter, Chess Boughey, Michael Smith, Sohini Kar‐Narayan

**Affiliations:** ^1^ Department of Materials Science University of Cambridge 27 Charles Babbage Road Cambridge CB3 0FS UK

**Keywords:** ferroelectric polymers, finite element modelling, nanogenerators, nanowires, template wetting

## Abstract

Nanowires of the ferroelectric co‐polymer poly(vinylidenefluoride‐*co*‐triufloroethylene) [P(VDF‐TrFE)] are fabricated from solution within nanoporous templates of both “hard” anodic aluminium oxide (AAO) and “soft” polyimide (PI) through a facile and scalable template‐wetting process. The confined geometry afforded by the pores of the templates leads directly to highly crystalline P(VDF‐TrFE) nanowires in a macroscopic “poled” state that precludes the need for external electrical poling procedure typically required for piezoelectric performance. The energy‐harvesting performance of nanogenerators based on these template‐grown nanowires are extensively studied and analyzed in combination with finite element modelling. Both experimental results and computational models probing the role of the templates in determining overall nanogenerator performance, including both materials and device efficiencies, are presented. It is found that although P(VDF‐TrFE) nanowires grown in PI templates exhibit a lower material efficiency due to lower crystallinity as compared to nanowires grown in AAO templates, the overall device efficiency was higher for the PI‐template‐based nanogenerator because of the lower stiffness of the PI template as compared to the AAO template. This work provides a clear framework to assess the energy conversion efficiency of template‐grown piezoelectric nanowires and paves the way towards optimization of template‐based nanogenerator devices.

## Introduction

Ferroelectric polymers^1^, ^2^, ^3^ are of scientific and technological interest as, unlike typical ferroelectric ceramics, they are flexible, light‐weight, bio‐compatible, and low‐temperature and solution‐processable, and are attractive for use in piezoelectric generators for mechanical energy harvesting. Ferroelectric/piezoelectric polymeric nanowires are commonly incorporated into “nanogenerators”,^4^, ^5^, ^6^ which have been found to outperform bulk or thin‐film devices, and these have been attracting increasing interest as energy solutions for small power devices such as portable electronics, wireless sensor nodes, biomedical implants, and structural monitoring devices. This interest is intensified by current technological trends such as the increasing prevalence of autonomous sensors, which have been predicted to rise rapidly, fueling the growth of the “Internet of Things”^7^ linking everyday objects.

**Figure 1:**
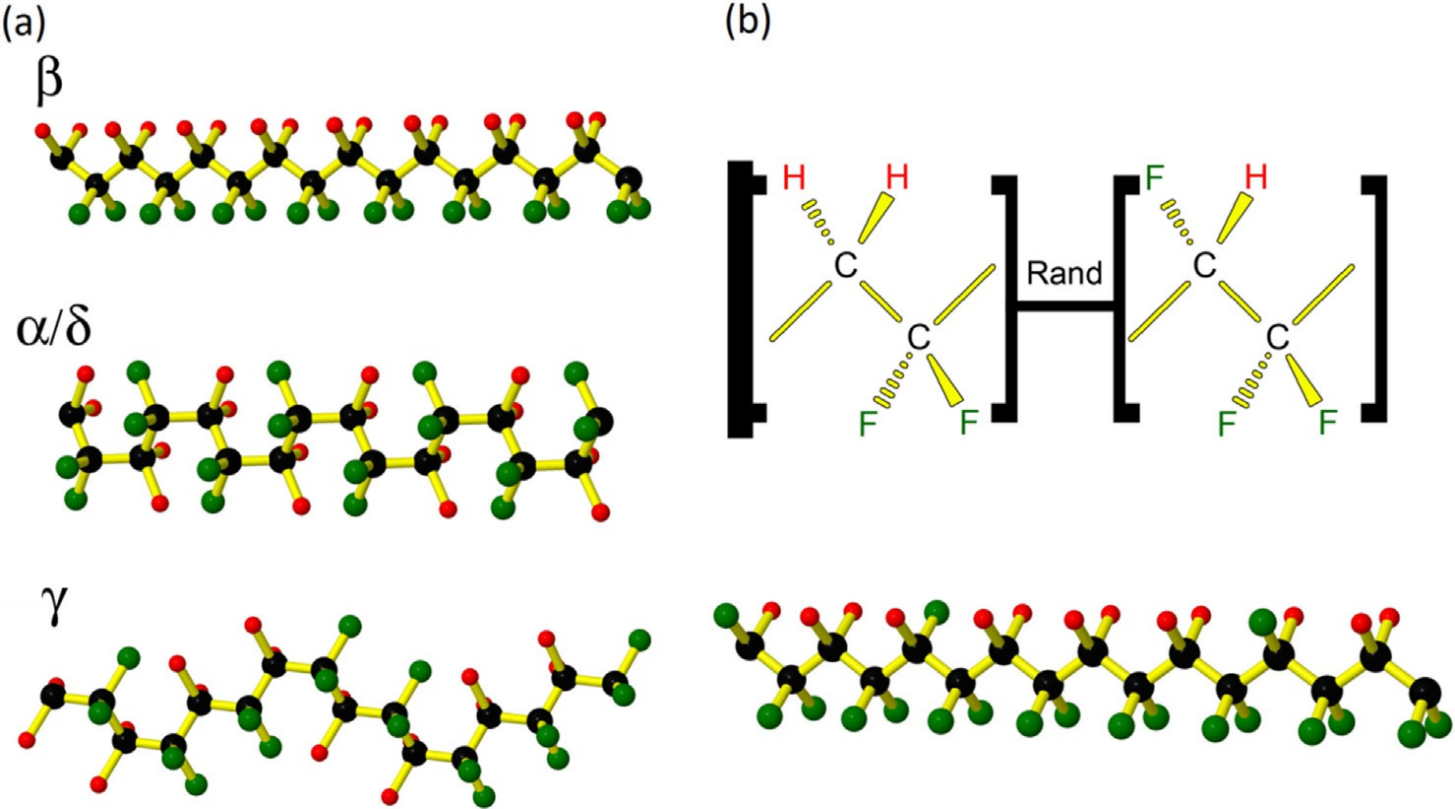
a) Chain conformations of PVDF for the indicated crystalline phases. Carbon atoms are depicted as black, fluorine as green, and hydrogen as red. Bonds are depicted in yellow. b) Monomer units and chain conformation of P(VDF‐TrFE).

While there have been recent reports of ferroelectric polymers, such as polyamides (odd‐numbered nylons),^8^, ^9^ having found applications in mechanical energy harvesting, polyvinylidine fluoride (PVDF) and its co‐polymers have received by far the most interest for ferroelectric polymer nanogenerator applications^4^, ^10^, ^11^, ^12^, ^13^, ^14^, ^15^, ^16^, ^17^, ^18^, ^19^ due to their superior electromechanical properties. PVDF is a fluoro‐polymer known to exhibit piezoelectricity since 1969^20^ and ferroelectricity since 1981.^21^ PVDF consists of a carbon backbone with each carbon in the chain alternatively binding two fluorine or two hydrogen atoms oriented on opposite sides of the carbon chain. The piezoelectric properties of PVDF arise from the large difference in electronegativity between the fluorine and carbon atoms compared with the hydrogen atoms. This results in polar bonds and a resulting dipole moment from the fluorine side of the chain towards the hydrogen side.^1^, ^3^, ^22^ Chain conformations for different crystalline phases of PVDF are shown in Figure 1 a. The β phase of PVDF is the all‐*trans* phase (TTTT), which has the highest spontaneous polarization^22^, ^23^ and is thus desirable for piezoelectric applications. This phase is more readily realized in P(VDF‐TrFE), which is a co‐polymer consisting of polymer chains alternating non‐periodically between vinylidene‐fluoride (VDF) and trifluoro‐ethylene (TrFE),^22^, ^24^, ^25^ as shown in Figure 1 b. We have previously demonstrated that P(VDF‐TrFE) nanowires grown via “template‐wetting” showed enhanced crystallinity, with a net polarization along the length of the nanowires causing them to be “self‐poled”.^10^, ^26^ The observed preferential crystallization behavior had earlier been attributed to preferential nucleation and growth from the pore walls during template infiltration.^12^, ^15^ In ref. 26, the self‐poled nature of the nanowires was explored in detail, revealing similar physical properties as electrically poled thin‐film samples of the same material. In the same study, piezoresponse force microscopy (PFM) was used to establish self‐poling in the nanowires, which was otherwise not observed in an unpoled as‐grown film. This is therefore particularly advantageous for piezoelectric applications involving template‐grown P(VDF‐TrFE) nanowires whereby an external electric poling field is not required.

Template‐wetting is a simple and scalable nanowire fabrication method that involves the infiltration of a polymer melt or solution into a nanoporous template,^12^, ^13^, ^15^, ^17^, ^27^, ^28^, ^29^, ^30^ followed by solidification or solvent evaporation giving rise to nanowires or nanotubes formed within the template. This process relies on the high surface energy of the template walls, as infiltration is predominantly driven by the difference in surface energy between pore walls and infiltrating polymer. We have previously shown that template‐wetting can be used to achieve self‐poled P(VDF‐TrFE) nanowires grown within both hard anodic aluminium oxide (AAO) templates (Young's modulus *Y*≈122 GPa^31^), as well as soft polyimide (PI) templates (*Y*≈3 GPa^32^, ^33^), where the choice of template material was shown to play a role in determining the crystallinity of the nanowires.^26^ Here, we report on the energy‐harvesting performance of nanogenerators that have been assembled from P(VDF‐TrFE) nanowires embedded in both AAO and PI templates in order to ascertain the role played by the template in determining nanogenerator output performance. Importantly, we present detailed computational modelling of these template‐based nanogenerators to assess the performance of the P(VDF‐TrFE) nanowires within the nanogenerator devices and, in particular, to determine their electromechanical conversion efficiency and relevant nanogenerator figures of merit.^6^ Our studies pave the way for device optimization involving any combination of template and ferroelectric/piezoelectric nanowire material, and a reliable means to predict mechanical energy‐harvesting performance at the nanogenerator design stage.

## Results and Discussion

**Figure 2:**
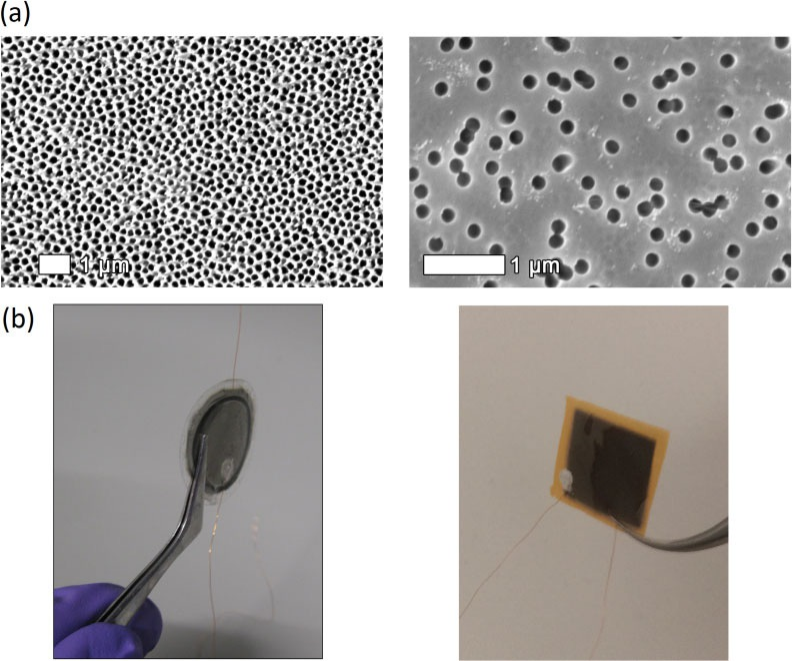
a) SEM images of bare AAO (left) and PI (right) template surfaces. b) Photographs of nanogenerators based on P(VDF‐TrFE) nanowire‐filled templates.

P(VDF‐TrFE) nanowires, of diameter 200 nm and lengths ≈60 μm and ≈20 μm, were fabricated within AAO and PI templates, respectively, via template‐wetting as described in detail in previous work.^10^ The pore sizes are nominally identical in both of these templates, although the AAO templates have higher porosity than the PI templates. Figure 2 a shows scanning electron microscopy (SEM) images of the bare templates prior to infiltration of the polymer to form nanowires, while Figure 2 b show photographs of nanogenerators based on the respective AAO and PI templates that were assembled by sputtering electrodes onto both sides and attaching wires for electrical access.

**Figure 3:**
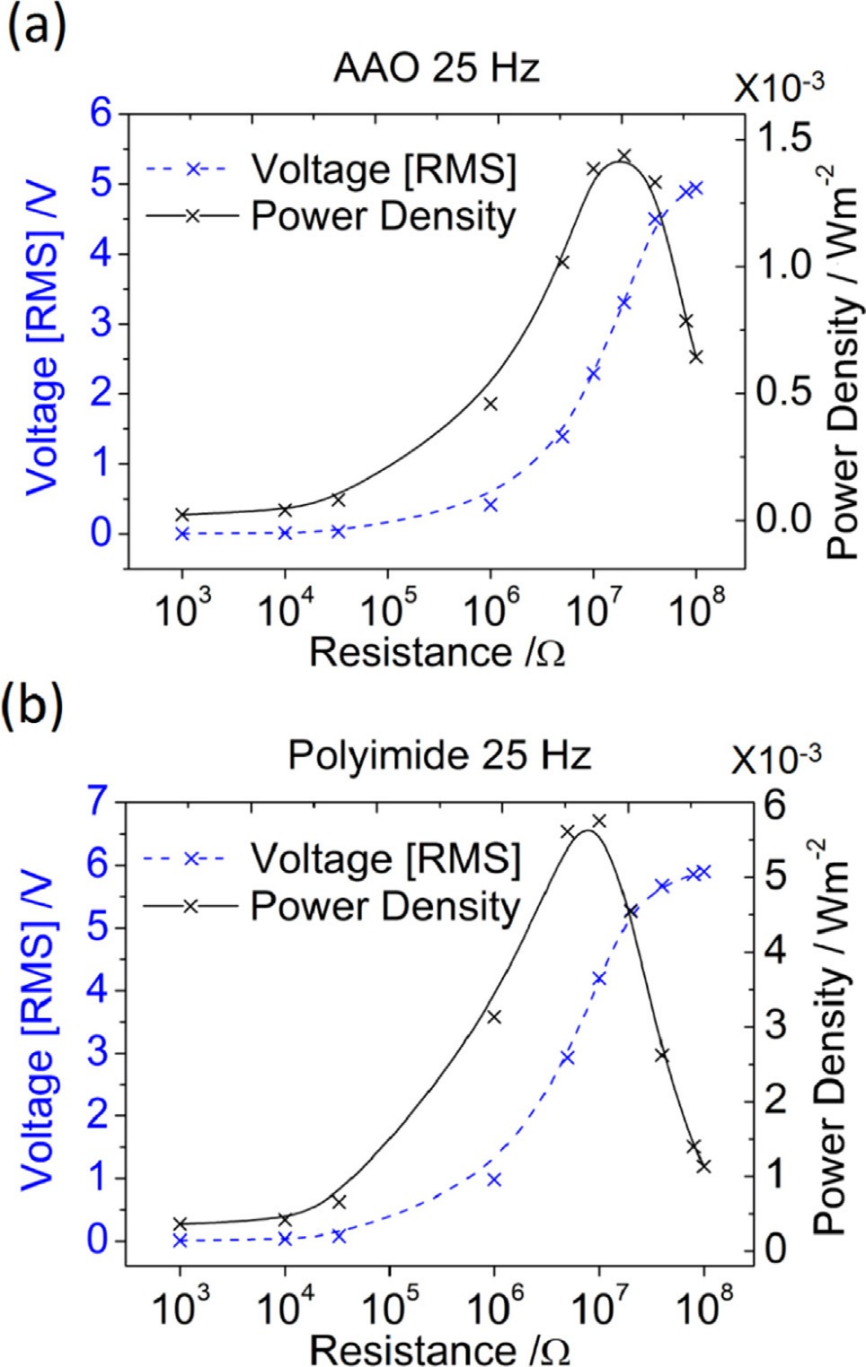
RMS voltage and normalized power density of a P(VDF‐TrFE) nanowire‐filled a) AAO template‐based nanogenerato and b) PI template‐based nanogenerator, as a function of load resistance.

Energy‐harvesting measurements were carried out in a bespoke setup (previously described in ref. 10) used to test the nanogenerator output performance in response to a periodic mechanical impacting excitation having frequency ranging from 5–25 Hz, and at a constant driving amplitude of 2 mm. Typical output voltage waveforms as measured for the AAO‐based and PI‐based nanogenerators at different frequencies are shown in Supporting Information S1. The output power is measured across a series of load resistances to determine the maximum power output across an impedance‐matched load. Figure 3 shows a representative graph depicting the variation of root mean square (RMS) voltage and power density of an AAO‐ and PI‐based device being impacted at 25 Hz, respectively, as a function of load resistance, where the power is determined by the square of the RMS voltage divided by the resistance. The RMS voltage values were determined from device output signals each of at least 5 s in duration for at least three devices prepared with identical methods. Current and voltage output from different devices successfully prepared with identical methods had standard errors of ≈6 % or less. Peaks in the power output for matched impedance loads were observed as expected, and have been reported for other energy harvesting piezoelectric devices using PVDF‐based polymers.^34^ The load that gives the maximum power output was found to decrease slightly with frequency and to be slightly less for the polyimide devices compared with the AAO devices. This varies from 40–20 MΩ for an AAO device and 20–10 MΩ for a polyimide device (see Supporting Information S2).

**Figure 4:**
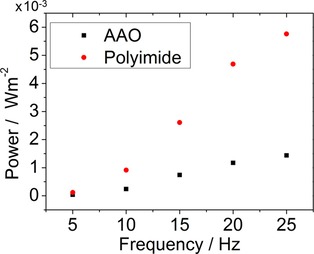
Output power densities as a function of frequency for P(VDF‐TrFE) nanowire‐filled AAO and PI template‐based nanogenerators.

Figure 4 shows a plot of peak power density for AAO and PI devices as a function of frequency. The peak output power density is higher in the PI‐based device than that of an AAO‐based device despite there being less piezoelectric material present as a result of the relative thicknesses and porosity of the templates. This is due to the fact that AAO is significantly stiffer than PI, which results in far less strain and effective stress in the P(VDF‐TrFE) nanowires within an AAO device compared to within a PI device.

In order to compare the performance of the two different template‐based devices and the template‐grown P(VDF‐TrFE) nanowires themselves, the device and material efficiency, and stress and strain figures of merit, *η*_T_ and *η*_S_,^6^ provide the most meaningful metrics. In order to determine these values, the mechanical input energy must be considered for the device as a whole, while for the material efficiency, only the component of the input energy that contributes to stress/strain in the P(VDF‐TrFE) nanowires themselves is relevant. To achieve this, we use computational modelling of the devices for further analysis and comparison.

For modelling the piezoelectric energy‐harvesting devices, a finite element method (FEM) was used, which is a well‐established technique for modelling 3D systems of arbitrary geometry and has been previously used to model piezoelectric nanostructures.^35^, ^36^, ^37^ Modelling was carried out within the software package COMSOL Multiphysics 5.2. Due to the scale mismatch between the macroscopic devices and their nanoscale features, modelling of a complete device was computationally impossible due to the required number of elements to adequately mesh the geometry. The approach taken was therefore to model small areas of a device containing a manageable number (≤61) of full‐length nanowires and to determine the potential difference created across the nanowires for a given stress applied to the device. Given the restriction on the area of device that could be modelled, this approach benefits from the fact that the nanowires act as parallel capacitors in the device geometry.^36^ This means that a stress applied uniformly across the top surface of the device, as was the case in our energy harvesting measurement setup, the potential difference across each nanowire is the same and that of a single nanowire is the same as the device as a whole. The results from the model could be compared with peak open‐circuit voltage measurements of the devices for different mechanical input frequencies (associated with different peak stresses). This approach of calculating applied stress and/or strain from open‐circuit voltage has been used analytically in other reported work on piezoelectric energy‐harvesting devices.^35^, ^37^ From the stress and strain calculated by the model, the strain energy of the nanowires can be calculated and then compared with the measured electrical energy output, in order to determine the material energy conversion efficiency of the respective template‐grown P(VDF‐TrFE) nanowires, as well as *η*_T_ *and η*_S_.^6^

**Figure 5:**
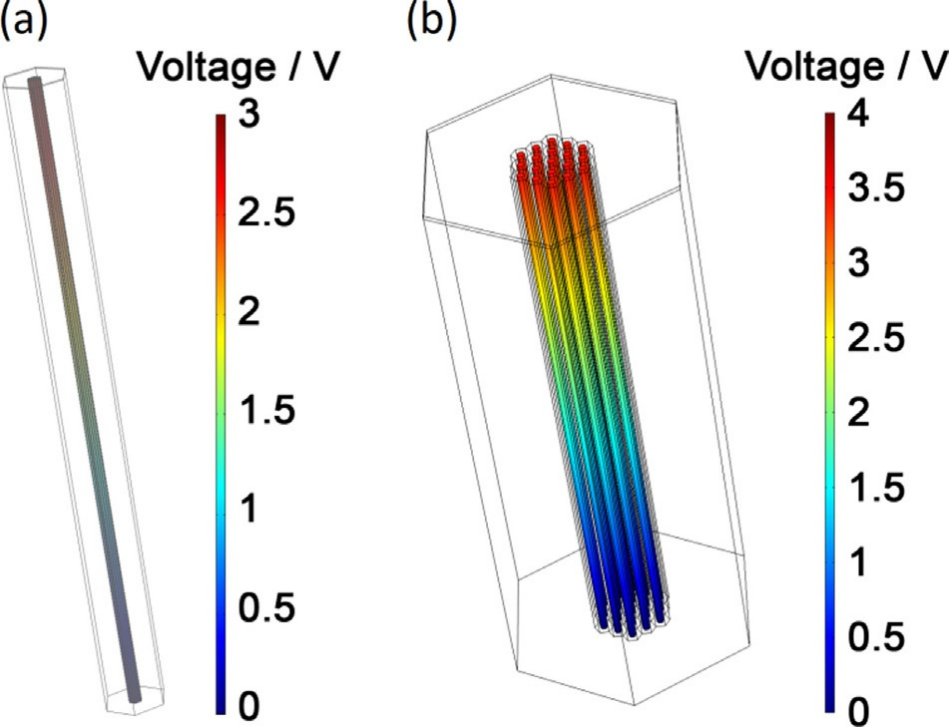
Examples of electrical potential distribution in nanowires from finite element models of a) AAO and b) PI‐based nanogenerator devices.

A detailed description of the model and parameters used is provided in Supporting Information S3. The values of the piezoelectric constant and Young′s modulus of the P(VDF‐TrFE) nanowires used in the model had been directly determined using piezoresponse force microscopy (PFM) and quantitative nanomechanical mapping (QNM), respectively, as reported previously.^26^, ^38^ Open‐circuit voltage measured using the energy harvesting set‐up was used in conjunction with the FEM models to determine applied axial stresses (see Supporting Table S3). Figure 5 shows examples of the electrical potential distribution in nanowires within models of an AAO and PI‐based device for the same level of mechanical excitation.

The strain energy of an isotropic material, *W*_S_, is given by Equation (1),^39^, ^40^ where *A* is the cross‐sectional area, *L* is the length (thickness) of the material, *T* is the stress and *S* is the strain. For calculation of the strain energy of one of the devices, the polydimethylsiloxane (PDMS) layers coating the top and bottom electrode surfaces needed to be considered in addition to the nanowire filled nanoporous template. The total strain energy of a device for each impact cycle, $W_{S}^{D}$ , was given by the summation of the strain energies of the template ($W_{S}^{T}$ ), nanowires ($W_{S}^{NW}$ ), and PDMS layers ($W_{S}^{PDMS}$ ), as shown in Equation (1).

(1) $$W_{S}=AL\int TdS=\frac{1}{2}ALTS$$

(2) $$W_{S}^{D}=W_{S}^{T}+W_{S}^{NW}+W_{S}^{PDMS}$$

The combined strain energy of the nanowire‐filled nanoporous template is given by Equation (3) where *A*_T_ is the cross‐sectional area of the filled template, *L*_T_ is the thickness of the template (also equal to the length of the nanowires), *T*_axial_ is the peak axial applied stress, and *S*_axial_ is the peak axial strain given by Equation (4) where *Y*_NW_ and *Y*_T_ are the Young's moduli of nanowires and template, respectively, and *p*_T_ is the porosity of the template.

(3) $$W_{S}^{T}+W_{S}^{NW}=\frac{1}{2}A_{T}L_{T}T_{axial}S_{axial}$$

(4) $$S_{axial}=\frac{T_{axial}}{p_{T}Y_{NW}+\left(1-p_{T}\right)Y_{T}}$$

For calculation of $W_{S}^{PDMS}$ , the axial strain in the PDMS layers, $S_{PDMS}$ , needed to be considered. As the PDMS layers were mechanically connected in series with the filled template (unlike the template and nanowires, which were mechanically connected in parallel) $S_{PDMS}$ differs from $S_{axial}$ . Because the PDMS layers were thin and adhered to the electroded template surface, the lateral strain of the PDMS was confined by the lateral strain in the template. $S_{PDMS}$ was therefore taken to be given by Equation (5) by equating the two lateral strains through introduction of the Poisson′s ratios of the template and PDMS, *ν*_T_, and *ν*_PDMS_. $W_{S}^{PDMS}$ could therefore then be given by Equation (6), where *L*_PDMS_ is the thickness of a PDMS layer and taking into account that there are two PDMS layers. From Equation (2), Equation (3) and Equation (6), $W_{S}^{D}$ may therefore be given by Equation (7).

(5) $$S_{PDMS}=\frac{\nu_{T}}{\nu_{PDMS}}S_{axial}$$

(6) $$W_{S}^{PDMS}=A_{T}L_{PDMS}T_{axial}S_{PDMS}=\frac{\nu_{T}}{\nu_{PDMS}}A_{T}L_{PDMS}T_{axial}S_{axial}$$

(7) $$W_{S}^{D}=A_{T}T_{axial}S_{axial}\left(\frac{1}{2}L_{T}+\frac{\nu_{T}}{\nu_{PDMS}}L_{PDMS}\right)$$

To determine the material energy conversion efficiency of the piezoelectric nanowires within a device, the strain energy of just the nanowires, $W_{S}^{NW}$ , needed to be calculated. This is given by Equation (8) where *A*_NW_ is the cross‐sectional area of the active nanowires determined from the electrode area and the porosity of the template.

(8) $$W_{S}^{NW}=\frac{1}{2}A_{NW}L_{T}Y_{NW}S_{axial}^{2}$$

The electrical energy generated by a device with each impact, *W*_E_, was determined from the integral of the product of current *I* and voltage *V* with respect to time *t* for one impact cycle, where *I* and *V* were measured across an impedance‐matched load for maximum power output. For each frequency and device, type values of *W*_E_ were determined by averaging over calculation from at least 30 cycles. From values of *W*_E_, device and material efficiencies, *χ*_D_ and *χ*_NW_,^6^ could then be determined using Equations (9), (10), respectively.

(9) $$\chi_{D}=\frac{W_{E}}{W_{S}^{D}}$$

(10) $$\chi_{NW}=\frac{W_{E}}{W_{S}^{NW}}$$

The predictive capability of the model was tested for a range of experimental parameters, as explained in Supporting Information S4. Table 1 shows values of $W_{S}^{NW}$ , *W*_E_, and *χ*_NW_ determined from the model in conjunction with experimental results for AAO and PI devices. The values of *χ*_NW_ for P(VDF‐TrFE) were seen to be consistent for each device type with average values of 7.1 % and 6.4 % for AAO‐ and PI template‐based devices, respectively. No clear frequency dependence was seen, which is expected for this device geometry^6^ where the frequencies used are far from expected resonances and therefore this further validates the determined values. Importantly, a lower value of *χ*_NW_ for P(VDF‐TrFE) nanowires grown in PI templates relative to those grown in AAO templates is consistent with materials characterization that was carried out and detailed in previous work,^26^ where lower crystallinity was reported for nanowires grown in PI templates as compared to those grown in AAO templates. The FEM analysis described here can be easily extended to other template‐grown polymeric materials^8^, ^41^ for energy harvesting applications.

**Table 1:** Table of nanowire strain energy, electrical energy and materials efficiency for AAO and PI‐based nanowires with impact frequencies of 5–25 Hz.

	Frequency [Hz]	*W*_S_^NW^ [nJ]	*W*_E_ [nJ]	*χ*_NW_ [%]
AAO	5	26.7	1.88	7.04
	10	92.4	6.67	7.22
	15	156	11.2	7.19
	20	234	16.2	6.92
	25	275	19.6	7.11
Polyimide	5	84.9	5.46	6.44
	10	300	19.0	6.34
	15	499	31.4	6.30
	20	722	47.6	6.59
	25	838	53.4	6.38

Interestingly, average values of *χ*_D_ for AAO and PI devices were found to be 0.10 % and 0.75 %, respectively, even though the PI template‐grown P(VDF‐TrFE) nanowires showed a lower *χ*_NW_ as compared to AAO template‐grown nanowires. This could be attributed to the higher stiffness of the AAO template as compared to the PI template, which meant that a larger fraction of the input mechanical energy was lost to deforming the AAO template than the PI template leading to overall lower device efficiency. In both cases the nanogenerator device design resulted in *χ*_D_ being low compared to *χ*_NW_, which represents a theoretical limit of *χ*_D_ for a given material. Additionally, figures of merit *η*_T_ and *η*_S_,^6^ were also determined, with values of 0.18 GJ m^−3^ and 28.4 pJ m^−3^ Pa^−2^ for the P(VDF‐TrFE) nanowires fabricated in AAO templates and values of 0.16 GJ m^−3^ and 25.6 pJ m^−3^ Pa^−2^ for P(VDF‐TrFE) nanowires fabricated in PI templates. In practice other considerations of device design need to however be considered to allow adequate integration, robustness, and reliability of devices.

## Conclusions

Template‐wetting has been shown to be an attractive fabrication method for ferroelectric/piezoelectric polymeric nanowires due to its simplistic nature that is not reliant on complicated set‐ups and the use of high temperatures and/or pressures. Importantly, the realization of self‐poled nanowires^8^, ^10^ through this method makes it particularly attractive in nanogenerator applications. In this work, we presented a combination of experimental results and computational modelling to determine primarily the role played by the template in the energy‐harvesting performance of P(VDF‐TrFE) nanowires fabricated within them via template‐wetting. While we had previously shown that the choice of template material determines the crystallinity of the ferroelectric polymer nanowires fabricated within the template,^26^ here we have shown how this also affects the piezoelectric energy‐harvesting capability of the nanowires. It was shown, both through direct measurements of electrical output in response to periodic impacting as well as through finite element modelling of the nanowire‐filled template‐based nanogenerator devices, that AAO template‐grown P(VDF‐TrFE) nanowires had higher energy conversion efficiency than their PI template‐grown counterparts, because of greater crystallinity. However, the overall device efficiency was higher for the PI template‐based device due to the lower stiffness of the PI template as compared to AAO template. The computational models developed in this work can be easily applied to any combination of ferroelectric/piezoelectric polymer and template material to predict the energy‐harvesting performance of template‐grown polymer nanowires, thus enabling ease of optimization of template‐based nanogenerator design for specific applications.

## Experimental Section

The two types of commercial nanoporous templates used in this work were AAO templates (Anapore, Whatman) and PI track‐etched templates (ipPore, it4ip), both with nominal pore diameter of ≈200 nm. The AAO templates were ≈60 μm thick and ≈25 mm in diameter, with nominal porosity of ≈50 %, while the polyimide templates were ≈15 μm thick with nominal porosity of ≈15.7 %. The PI templates were purchased in the form of A4 sheets and cut to tiles typically of size 20 mm×20 mm for this work.

To produce the solutions in the template‐wetting process, P(VDF‐TrFE) in powder form with a molar composition of 70:30 of VDF:TrFE (Piezotech, France) was dissolved in methyl ethyl ketone (MEK) (Sigma–Aldrich), sonicated for ≈60 min, and then drop‐cast onto the templates. It was found that for the AAO and PI templates using solutions with a concentration of 10 % and 6 % by weight, respectively, and a hot plate temperature of 60 °C in both cases resulted in the formation of nanowires completely filling the length of the template pores.

Piezoelectric energy harvesting nanogenerators were assembled from as‐grown P(VDF‐TrFE) nanowire‐filled AAO and PI templates. Prior to the polymer infiltration of the templates, a thin film of platinum of thickness ≈100 nm was sputter coated (Emitech k550) on the bottom side of the template. Following polymer nanowire infiltration from the opposite side and subsequent removal of the residual polymer film as previously described, a thin film of platinum (≈100 nm) was deposited on the on top resulting in forming two electrodes on either side of the template contacting the enclosed nanowires. A shadow mask was used during sputter coating of the platinum electrodes, which defined the effective area of the device; this also prevented the platinum coating to the edge of the template, which in turn prevented electrical shorting of the device. Thin copper wires were then attached to each platinum layer using silver conductive paint (H K Wentworth) to allow for electrical access to the nanogenerator. For protection and to assist in stress being applied evenly across the devices during operation, the devices were coated in polydimethylsiloxane (PDMS).

## Conflict of interest

*The authors declare no conflict of interest*.

## Supporting information

As a service to our authors and readers, this journal provides supporting information supplied by the authors. Such materials are peer reviewed and may be re‐organized for online delivery, but are not copy‐edited or typeset. Technical support issues arising from supporting information (other than missing files) should be addressed to the authors.

Supplementary
