# Supplementary material for: Mechanical Energy Harvesting Performance of Ferroelectric Polymer Nanowires Grown via Template‐Wetting
Source: Energy Technol (Weinh). 2018 Feb 16;6(5):928–34. doi: 10.1002/ente.201700820 (PMC5993231; doi:10.1002/ente.201700820)
Supplement: Supplementary file 1 — Supplementary [file ENTE-6-928-s001.pdf]

Energy Technology

Supporting Information

Mechanical Energy Harvesting Performance of Ferroelectric Polymer Nanowires Grown via Template-Wetting

Richard A. Whiter, Chess Boughey, Michael Smith, and Sohini Kar-Narayan*[a]

ente_201700820_sm_miscellaneous_information.pdf

Supporting Information

Mechanical energy harvesting performance of ferroelectric polymer nanowires grown by template-wetting

*Richard A. Whiter, Chess Boughey, Michael Smith and Sohini Kar-Narayan**

S1. Voltage output of a P(VDF-TrFE) nanowire-filled template-based nanogenerators at different impacting frequencies

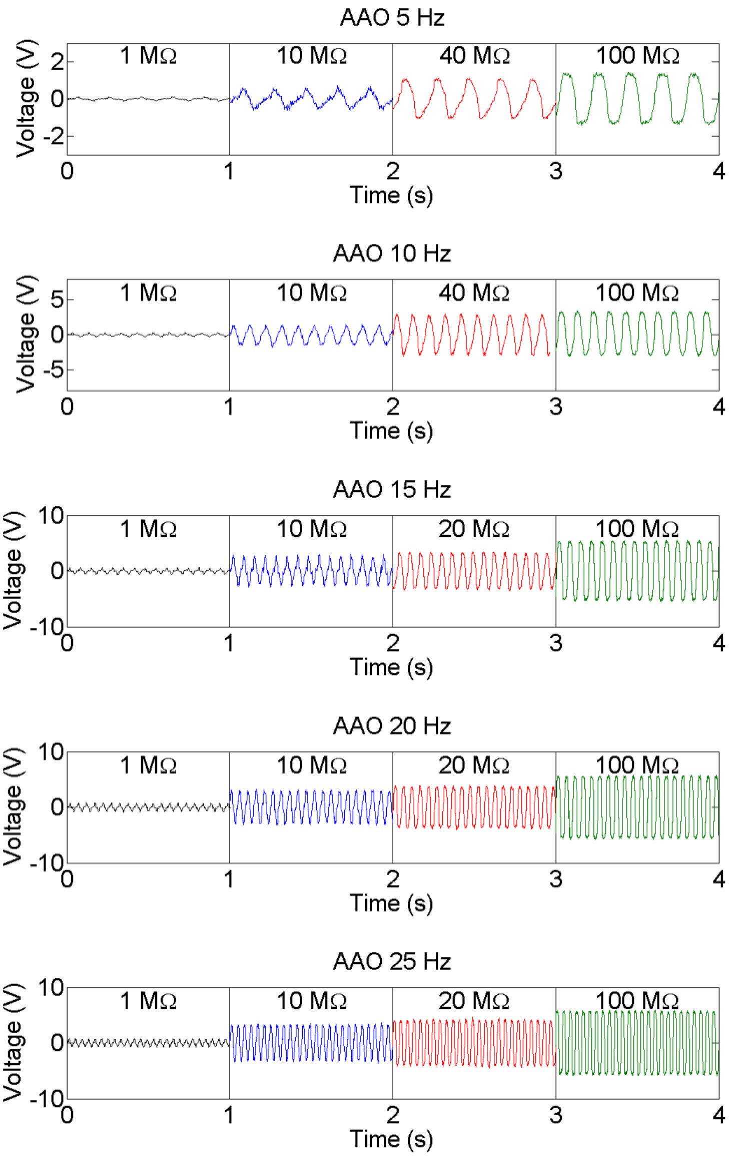

Figure S1: Voltage output of a P(VDF-TrFE) nanowire filled nanoporous AAO device at five different frequencies; 5 Hz, 10 Hz, 15 Hz, 20 Hz and 25 Hz. For each frequency measurement is shown for four different load resistances.

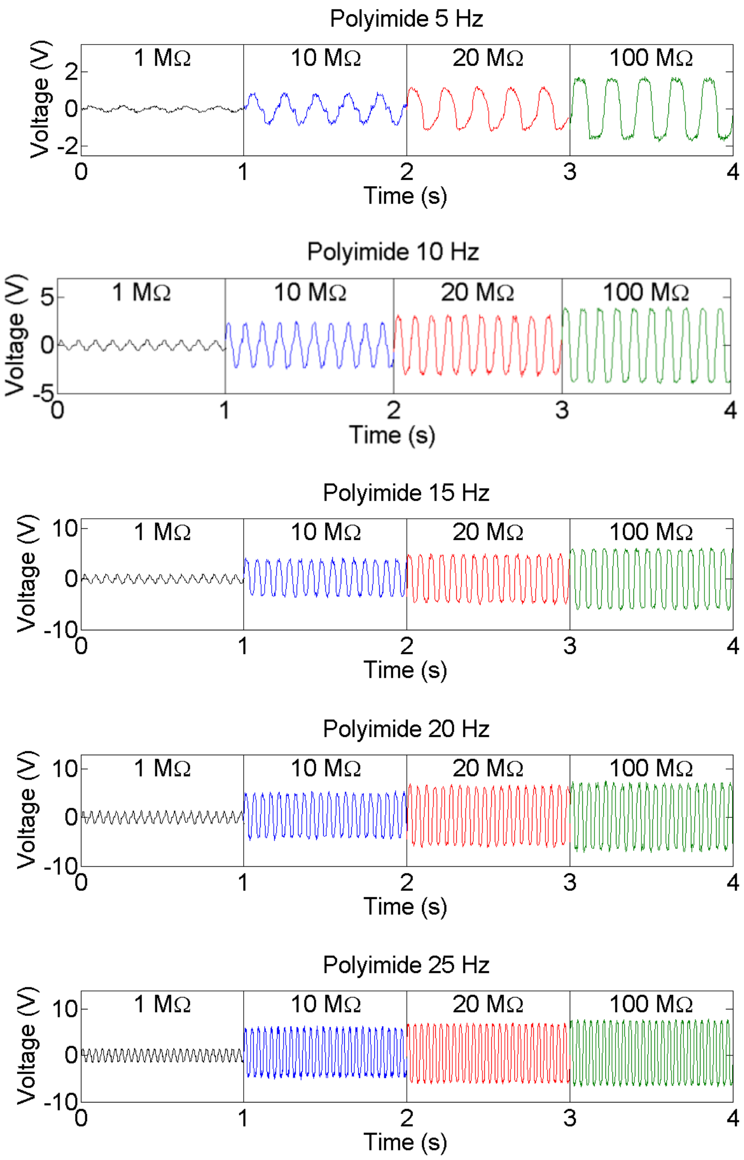

Figure S2: Voltage output of a P(VDF-TrFE) nanowire filled nanoporous PI device at five different frequencies; 5 Hz, 10 Hz, 15 Hz, 20 Hz and 25 Hz. For each frequency measurement is shown for four different load resistances.

S2. RMS voltage and normalised power density

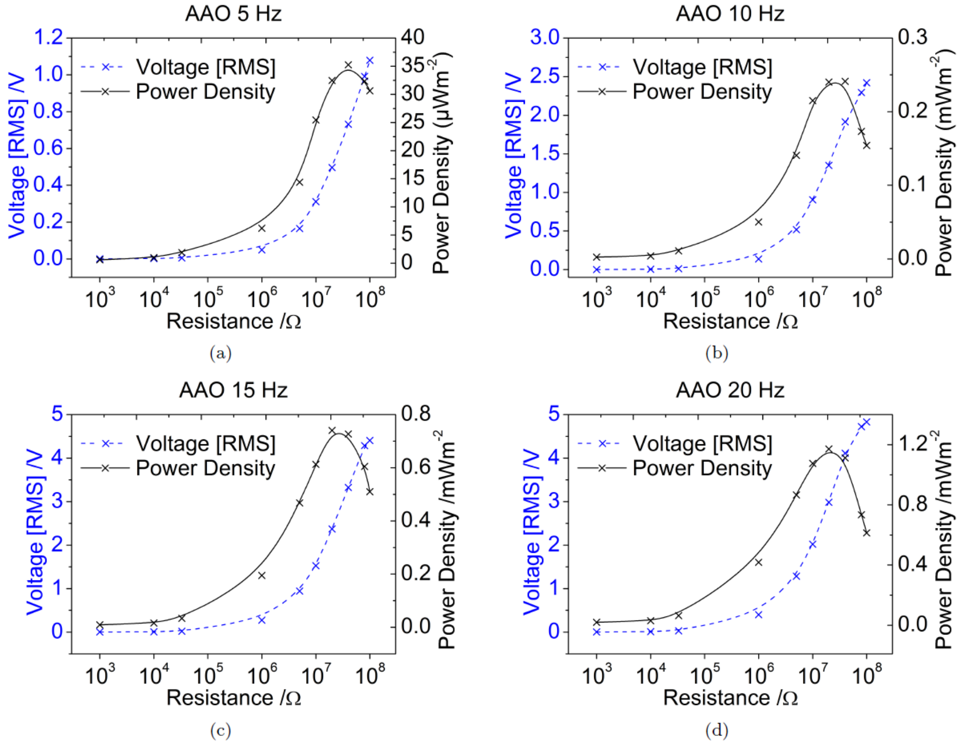

Figure S3: RMS voltage and power density of a P(VDF-TrFE) nanowire filled nanoporous AAO device as a function of the load resistance for different frequencies.

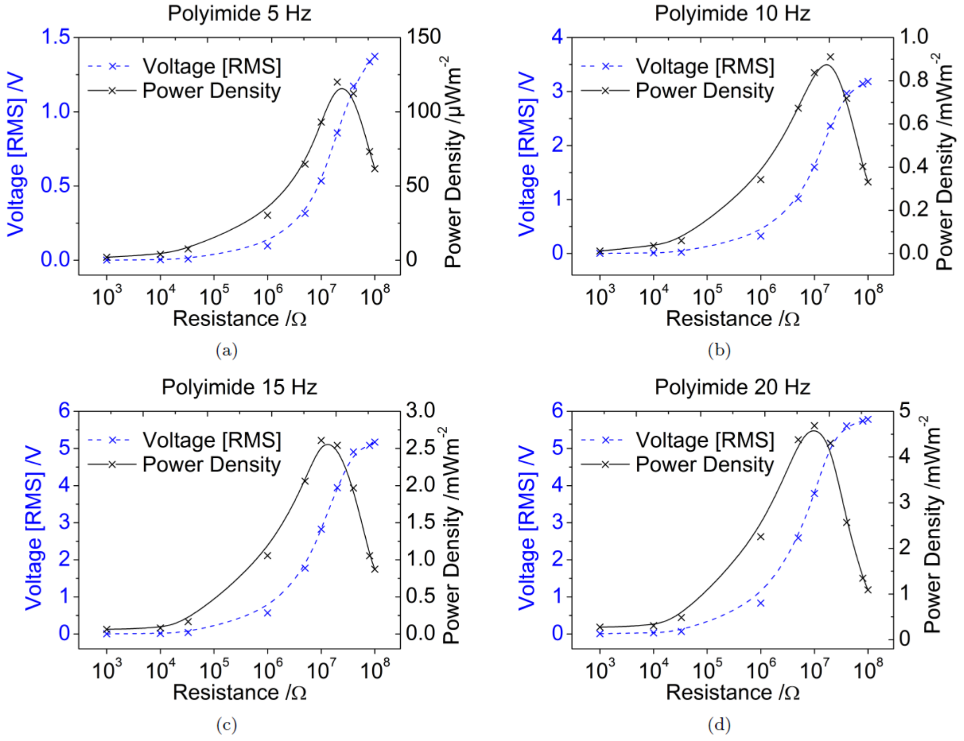

Figure S4: RMS voltage and power density of a P(VDF-TrFE) nanowire filled nanoporous PI device as a function of the load resistance for different frequencies.

S3: Finite element modelling of nanowire-filled template-based nanogenerators

<u>Geometry and Boundary Conditions</u>

The geometry of the modelled devices was based on arrays of hexagonal prisms representing the nanowires enclosed within larger hexagonal prisms representing the template, these were then both enclosed within a larger single hexagonal prism of a material representing a weighted average of the nanowires and template collectively. An example of this geometry is shown in Figure S5. The modelling could be carried out for different array sizes of the nanowires. For a cross-sectional plane of the model geometry, starting from a single nanowire hexagon representing an array size, n_{array}, of 1, larger array sizes were then taken to be given by the addition of successive concentric rings of nanowire and template hexagons. This is depicted in Figure S6 for values of n_{array} of 1, 2 and 3.

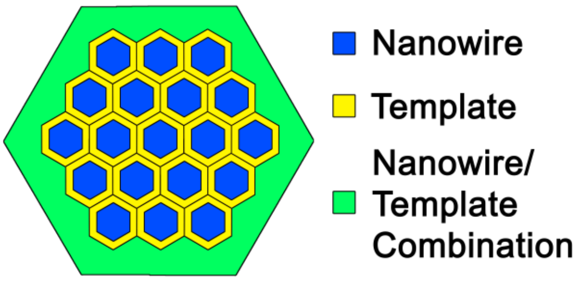

Figure S5: Diagram of the planar geometry used for modelling energy harvesting devices of piezoelectric nanowires inside nanoporous templates.

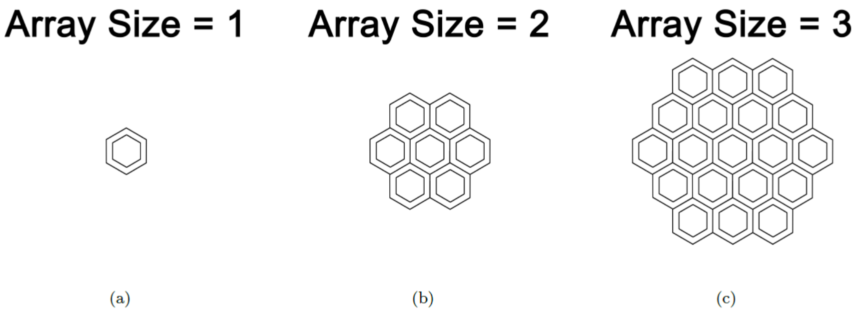

Figure S6: Diagrams depicting the planar geometry for array sizes of (a) 1, (b) 2 and (c) 3 in modelling energy harvesting devices.

The geometry within the model was defined within a two-dimensional plane starting with a single nanowire and template hexagonal pair. The side length of the nanowire hexagon, a_{NW}, was taken to be 100 nm as this is the nominal pore radius for both the AAO and polyimide templates. The side length of the template hexagon, a_T, was determined from the nominal porosity of the templates, p_T , being equated to the fractional area of the nanowire and template hexagons, given in Equation S1, which led to a_T being given by Equation S2.

$$p_T = \frac{3\sqrt{3}{a_{NW}}^2}{2} \Big/ \frac{3\sqrt{3}{a_T}^2}{2} = \frac{{a_{NW}}^2}{{a_T}^2} \tag{S1}$$

$$a_T = \frac{a_{NW}}{\sqrt{p_T}} \qquad \text{(S2)}$$

Additional parameters of x_{NW} and x_T were defined as depicted in Figure S7a and given by equation S3, as well as d_{NWT} which as depicted represents the difference between x_{NW} and x_T . Array sizes larger than 1 were created by first duplicating the central nanowire and template hexagons as appropriate and offsetting laterally by positive and negative integer multiples of a quantity defined as D_L shown in Figure S7b and given in Equation S4. This formed a row of nanowire and template hexagons, the complete array was then created by duplicating each nanowire and template hexagonal pair as appropriate and offsetting by positive and negative integer multiples of quantities defined as D_X and D_Y shown in Figure S7b and given by Equation S5 and Equation S6.

$$x_{NW} = \frac{\sqrt{3}}{2} a_{NW} \ ; \ x_T = \frac{\sqrt{3}}{2} a_T \qquad \text{(S3)}$$

$$D_L = 2(x_{NW} + d) \qquad \text{(S4)}$$

$$D_X = x_{NW} + d = x_T \qquad \text{(S5)}$$

$$D_Y = \sqrt{3}(x_{NW} + d) \qquad \text{(S6)}$$

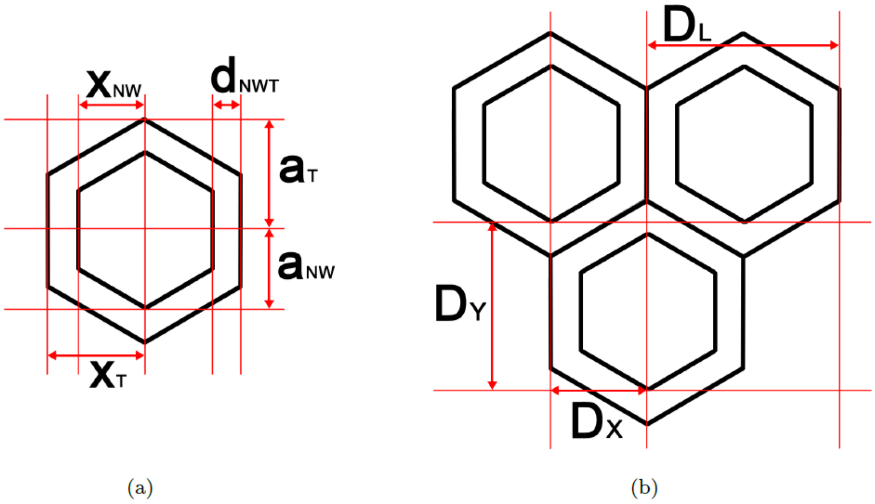

Figure S7: Diagrams depicting geometry parameters used in the models of piezoelectric energy harvesting devices.

The side length of the surrounding hexagon, a_{mat}, representing a combination of the nanowires and template was given by Equation S7 and as depicted in Figure S5, this hexagon was rotated 90° relative to the nanowire and template hexagons. Increasing the size of this hexagon beyond this point in all cases affected the output result of the model by <0.5%. The three-dimensional geometry was created by extruding the two-dimensional plane according to thickness of the template, 60 µm for AAO devices and 15 µm for polyimide devices. An additional rigid component of 100 nm thickness of the

same cross-section as the rest of the model was added to the top surface of the device geometry to which the stress was applied. The model was trialled with the addition of 10 μm thick PDMS components at the top and bottom of the device geometry, using the in-built software material properties for PDMS (Young's Modulus 750 kPa, density 970 kg/m^3, Poisson's ratio 0.49). The stress was then applied via the top surface of the top PDMS layer, this however resulted in all cases to affect the output result of the model by <0.3%, which can be expected by it not changing the stress applied in series through the geometry, and was therefore omitted to reduce computational time. The presence of the PDMS is however taken into account for calculation of the strain energy as explained in the main text.

$$a_{mat} = \frac{2}{\sqrt{3}}(x_T + 2x_T(n_{array} - 1) + 2\ \mu m) \tag{S7}$$

Boundary conditions were set for the device by introducing a fixed constraint condition which set the bottom surface of the device to be stationary. The bottom surface of the nanowires was set to an electrical potential of 0 (ground) to define a fixed potential point and allow a potential difference to be determined across the nanowires lengths.

Materials

Material properties were defined for the different components of the model. Starting with in-built material library properties for the relevant materials from the software, these properties were then modified based on measured values or those acquired from the literature. Table S1 shows the material properties of Young's modulus, Y, density, ρ, and Poisson's ratio, ν, for the materials used within the models. The properties of the material for the region surrounding the template and nanowire hexagons was determined from a weighted average of the nanowire and template material properties using the nominal porosity of the templates of 0.5 and 0.157 for the AAO and polyimide templates respectively. Compliance matrices were determined from Hooke's law for isotropic materials, taking the form shown in Figure S8.

**Material**	**Young's Modulus (GPa)**	**Density (kgm^{-3})**	**Poisson's Ratio**	**References**
AAO	122	3100	0.24	[1-5]
Polyimide	3.1	1400	0.34	[6-9]
P(VDF-TrFE)	2.73	1900	0.45	[10-12]

Table S1: Material properties used in modelling piezoelectric energy harvesting devices.

$$\frac{1}{Y}\begin{pmatrix} 1 & -\nu & -\nu & 0 & 0 & 0 \\ -\nu & 1 & -\nu & 0 & 0 & 0 \\ -\nu & -\nu & 1 & 0 & 0 & 0 \\ 0 & 0 & 0 & 1+\nu & 0 & 0 \\ 0 & 0 & 0 & 0 & 1+\nu & 0 \\ 0 & 0 & 0 & 0 & 0 & 1+\nu \end{pmatrix}$$

Figure S8: Compliance matrix of isotropic material

The model was trialled where the surrounding material was also treated as piezoelectric, with properties weighted to account for the fractional P(VDF-TrFE) content based on the porosity. In all cases however

this was found to affect the potential difference across the nanowires for a given applied stress by <1% and was therefore omitted to reduce computation time.

Verification of the model

To verify that the output of the model was valid for a full device the models were run with increasing array size to determine any trend in the change of the generated potential difference for a given stress. Figure S9 shows the percentage change in the generated potential difference for a given stress (which were found to be linearly related) for array size increases of consecutive values of n_{array} between 1 and 5. Data is shown for 5 different mesh sizes, with associated parameters given in Table S2 and shown in Figure S10. In both cases of AAO and polyimide templates, a larger percentage change was seen for an array size change from 1 to 2 (1-2%), with percentage changes then dropping to <0.5% and showing a trend of decreasing change towards 0 for larger array sizes. These low percentage changes and downward trend approaching 0 validate that the model, with relatively small array sizes, could be made applicable for a full device representing very large array sizes, tending towards infinity.

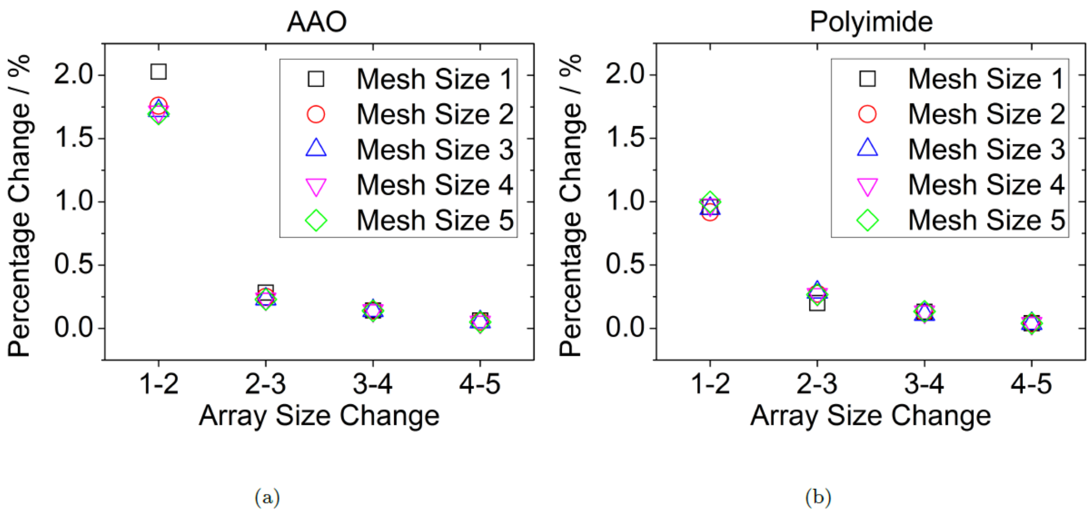

Figure S9: Plots of percentage change in potential difference generated for a given applied stress for consecutive increases in array size between 1 and 5 for (a) AAO and (b) polyimide devices.

Mesh Size #	Maximum Element Size / nm	Minimum Element Size / nm	Maximum Element Growth Rate
1	5000	120	2.00
2	3000	100	1.80
3	2200	90	1.65
4	1800	50	1.50
5	1200	20	1.30

Table S2: Mesh size parameters used in modelling piezoelectric energy harvesting devices. Additional parameters of curvature factor and resolution of narrow regions were used with values of 0.3 and 0.7 respectively in all cases.

In addition to checking dependence on array size, to verify that the output of the model was not dependent on mesh size, the models were run with 5 different mesh sizes with varying parameters shown in Table S2. In all cases a swept mesh was used with additional parameters of curvature factor of 0.3 and resolution of narrow regions of 0.7. Figure S10 shows images of the resulting meshes used for a model of an AAO device with an array size of 3. Figure S11 shows plots of percentage change in the potential difference generated for an applied stress for consecutive increases in the mesh sizes between

1 and 5. The percentage change is seen to be small, <0.1% for mesh sizes >2, and tends towards 0 for larger mesh size numbers corresponding to finer meshes.

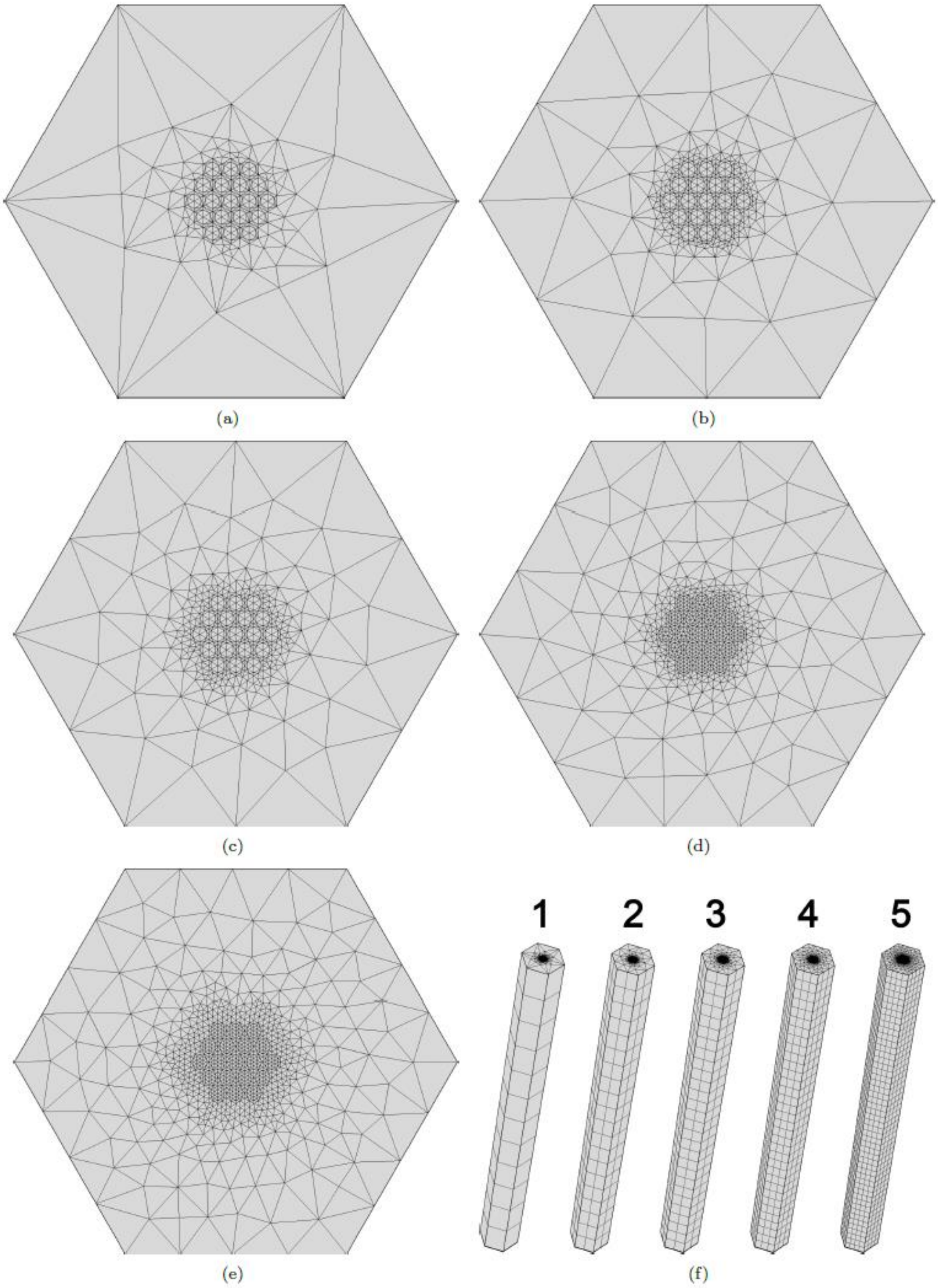

Figure S10: Images of different mesh sizes with the relevant parameters given in Table S2. (a)-(e) show the cross-sectional mesh for sizes 1-5 respetively. (f) shows meshes across the length of the geometry with the associated mesh size number given above each image.

Overall these verification tests based on array and mesh size indicated that the output of the model does not have a strong dependence on either and that any dependence tends towards 0 for both increasing array size and finer mesh sizes. This is important in demonstrating that the model used is valid for real devices with effective array and mesh sizes that could not be modelled directly due to limitations in computational power.

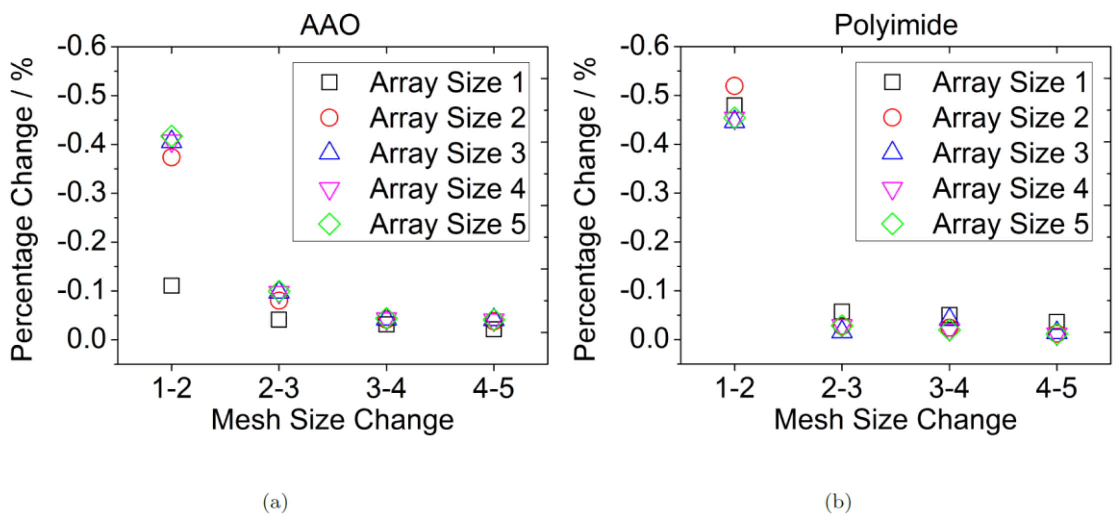

Figure S11: Plots of percentage change in potential difference generated for a given applied stress for consecutive increases in mesh size between 1 and 5 for (a) AAO and (b) polyimide devices.

Model Outputs

	Frequency / Hz	V_{OC} / V	Axial Stress / MPa	Axial Strain / 10^{-5}
	5	2.95	2.58	4.14
	10	5.49	4.80	7.70
**AAO**	15	7.13	6.24	10.0
	20	8.73	7.64	12.3
	25	9.47	8.29	13.3
	5	3.43	0.893	29.4
	10	6.45	1.68	55.2
**Polyimide**	15	8.32	2.16	71.2
	20	10.0	2.61	85.7
	25	10.8	2.81	92.3

Table S3: Open circuit voltage and axial stress and strain determined with FEM models.

S4: Predictive Capability of the Model

To ensure that the models used have predictive capability for proposed alternative materials and/or geometries, known values of W_S^D (see main text) are required. As the same mechanical input was used for measurement of different devices within the energy harvesting set-up, the W_S^D values may be expected to be the same independent of device.

Calculated values of W_S^D for AAO and polyimide devices are plotted for the five frequencies of measurement in Figure S12. As seen in Figure S12, all values were the correct order of magnitude relative to each other with close correlation, particularly for the lower frequencies, however for higher frequencies the values from the two types of devices were seen to, while showing the same characteristic trend, begin to increase in difference. A possible reason to account for the difference in values was

errors in parameters used within the calculation giving rise to an errant factor. They could alternatively be potentially accounted for by the mechanical energy supplied from the shaker more efficiently passing into the AAO devices compared with the polyimide devices but there was no reason determined to expect this.

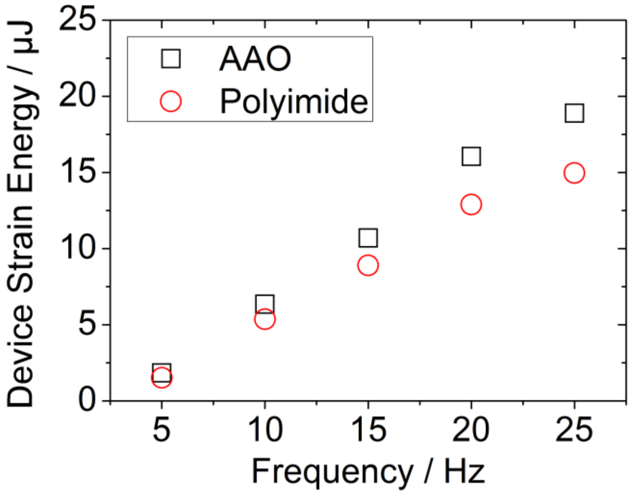

Figure S12: Plots of device strain energy against impact frequency for AAO and polyimide devices.

Despite the differences, as the values of W_S^D for AAO and polyimide devices remain close, prediction from the model with reasonable margin or error are still anticipated to be possible. This case is strengthened by the fact that the results for AAO and polyimide are from templates with material properties, such as stiffness, at either end of the typical range of template materials that may be considered. Nevertheless, for greater confidence in prediction from the model for measurement of proposed alternative materials and/or geometries, measurement of a wider range of different materials and geometries would be beneficial which may be considered in future work.

To demonstrate predictive capability of open circuit voltage from the model Figure S13 shows a plot of experimental V_{oc} values measured for polyimide devices alongside values which were calculated by the model using values of W_S^D obtained from the AAO devices, percentage differences of ~10% were found for each frequency.

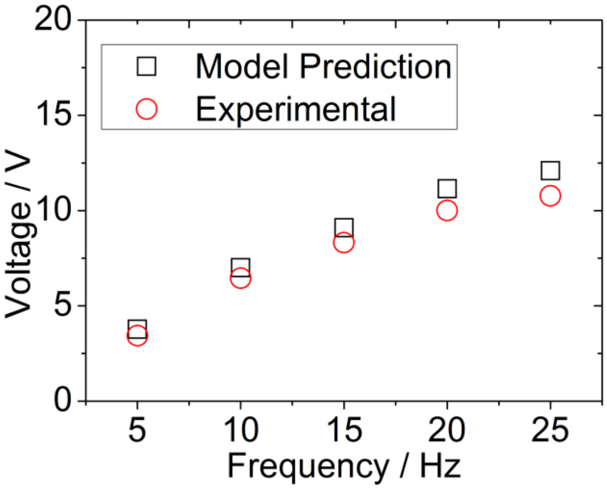

Figure S13: Plots of open circuit voltage experimental values and FEM model predicted values for a polyimide device with impact frequencies 5-25 Hz.

References:

1. X. Jiang, Y. Bin, and M. Matsuo, “Electrical and mechanical properties of polyimidecarbon nanotubes composites fabricated by in situ polymerization”, *Polymer*, vol. 46, no. 18, pp. 7418-7424, 2005.
2. W. M. Haynes, *CRC Handbook of Chemistry and Physics*. CRC Press, 2013. 94th Edition.
3. G. E. J. Poinern, N. Ali, X. Le, and D. Fawcett, “Nano-hardness and elastic modulus of anodic aluminium oxide based poly (2-hydroxyethylmethacrylate) composite membranes”, *AIMS Materials Science*, vol. 1, no. 20140303, pp. 159-173, 2014.
4. T.-H. Fang, T. Wang, C.-H. Liu, L.-W. Ji, and S.-H. Kang, “Physical behavior of nanoporous anodic alumina using nanoindentation and microhardness tests”, *Nanoscale Research Letters*, vol. 2, no. 8, pp. 410-415, 2007.
5. S. Ko, D. Lee, S. Jee, H. Park, K. Lee, and W. Hwang, “Mechanical properties and residual stress in porous anodic alumina structures”, *Thin Solid Films*, vol. 515, no. 4, pp. 1932 - 1937, 2006.
6. T. Furukawa and N. Seo, “Electrostriction as the origin of piezoelectricity in ferroelectric polymers”, *Japanese Journal of Applied Physics*, vol. 29, no. 4R, p. 675, 1990.
7. M. Stoppa and A. Chiolerio, “Wearable electronics and smart textiles: A critical review”, *Sensors*, vol. 14, no. 7, p. 11957, 2014.
8. R. A. Whiter, V. Narayan, and S. Kar-Narayan, “A scalable nanogenerator based on self-poled piezoelectric polymer nanowires with high energy conversion efficiency," *Advanced Energy Materials*, vol. 4, no. 18, p. 1400519, 2014.
9. M. C. Wang and B. D. Gates, “Directed assembly of nanowires”, *Materials Today*, vol. 12, no. 5, pp. 34- 43, 2009.
10. T. Furukawa, “Ferroelectric properties of vinylidene fluoride copolymers”, *Phase Transitions*, vol. 18, no. 3-4, pp. 143-211, 1989.
11. R. A. Whiter, Y. Calahorra, C. Ou, and S. Kar-Narayan, “Observation of confinement-induced self-poling effects in ferroelectric polymer nanowires grown by template wetting," *Macromolecular Materials and Engineering*, vol. 301, no. 9, pp. 1016-1025, 2016.
12. K. Thorkelsson, P. Bai, and T. Xu, “Self-assembly and applications of anisotropic nanomaterials: A Review”, *Nano Today*, vol. 10, no. 1, pp. 48 - 66, 2015.
